# Soil contamination by Taenia solium egg DNA in rural villages in Kongwa district, Tanzania

**DOI:** 10.1080/20008686.2020.1772668

**Published:** 2020-06-04

**Authors:** Justine Daudi Maganira, Winifrida Kidima, Chacha John Mwita, Peter Halvarsson, Johan Höglund

**Affiliations:** a Department of Biosciences, Sokoine University of Agriculture, Morogoro, Tanzania; b Department of Biomedical Sciences and Veterinary Public Health, Swedish University of Agricultural Sciences, Uppsala, Sweden; c Department of Zoology and Wildlife Conservation, University of Dar Es Salaam, Dar Es Salaam, Tanzania; d Department of Aquatic Sciences and Fisheries Technology, University of Dar Es Salaam, Dar Es Salaam, Tanzania

**Keywords:** *Taenia solium*, eggs, soil contamination, ddPCR, Kongwa, Tanzania

## Abstract

The presence of*Taenia solium* DNA from eggs in soils around the households in four Tanzanian villages in Kongwa district were analysed in relation to seasonal fluctuations and infection risk implications. A total of 192 pooled soil samples from five sampling points per household were examined by droplet digital Polymerase Chain Reaction (ddPCR) from 96 pig-keeping households both during the dry and rainy seasons. The pooled samples were first processed by a flotation-double sieving technique, followed by screening for worm DNA employing universal primers targeting the mitochondrial cytochrome c oxidase subunit I (*cox1*) gene of human taeniid species and some other helminths. All DNA positive samples were later confirmed by a specific ddPCR probe assay targeting the mitochondrial *cox1* gene of *T. solium*. A total of 17.2% (n = 33) samples were positive with the universal ddPCR, whereas *T. solium* DNA was confirmed by the specific ddPCR only in 3.1% (n = 3) of the surveyed households. The detection of *T. solium* DNA in this study spells out a low risk of exposure to *T. solium* eggs from contaminated household soil. Based on our results, ddPCR seems to be a promising technology for screening *T. solium* eggs in soil.

## Graphical abstract

Graphical Abstract

**Figure:**
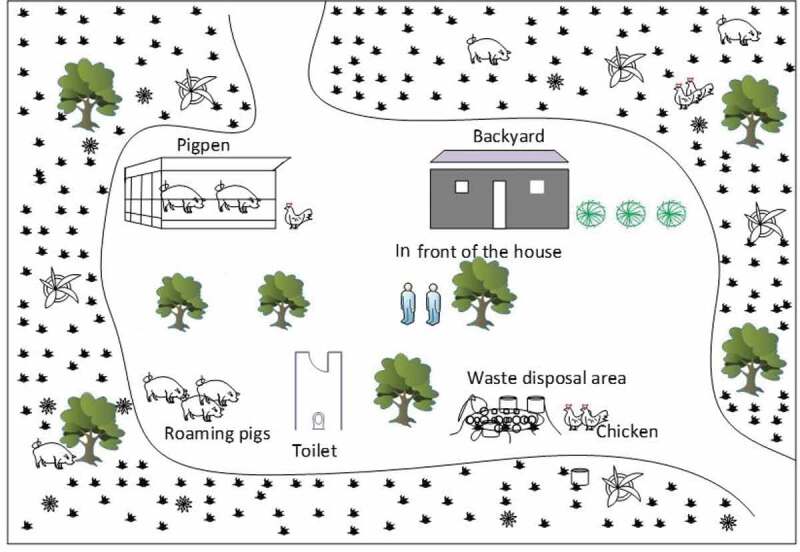

## Introduction

The cestode *Taenia solium* is a zoonotic parasite infecting both humans and pigs in developing countries of Asia, Latin America and Sub-Saharan Africa [1,2]. Humans act as the definitive host while pigs serve as the natural intermediate host [3]. It is well known that humans acquire taeniasis following consumption of the larval form of *T. solium* (cysticerci) in raw or inadequately cooked pork. In the human intestine, cysticerci develop into adult tapeworms that produce eggs, which accordingly can infect pigs. However, humans may also become accidental intermediate dead-end hosts either directly from the ingestion of eggs in human faeces or indirectly from eggs in the environment resulting in cysticercosis [3]. The most important form of cysticercosis infection in human is neurocysticercosis affecting the brain, which is reported as the main cause of acquired epilepsy in endemic regions [4].

The infection cycle of *T. solium* is usually associated with poor sanitary and hygiene conditions, free-range pig husbandry and inadequate or lack of meat inspection [5,6]. The prevalence of *T. solium* infection in Tanzania and elsewhere in Sub-Saharan Africa has been reported to range between 2.0% to 56.7% in pigs and 0.1% to 21.6% in humans [7], where they impact on the economies and nutritional well-being of rural communities. Reports are based on tongue palpation, conjunctival examination or meat inspection of pigs [2,8] and serological screening of both humans and pigs [5,9–12]. Application of coprological examination and computed tomography scanning methods, are restricted to the screening for *T. solium* infection in humans [10,11,13]. Meaningful programs to control cysticercosis cannot be adopted unless details in the infection cycle of taeniasis-cysticercosis are fully understood.

It is clear that people harbouring the adult *T. solium* have the potential for contaminating the environment with the parasite eggs when defecating in open areas. However, a most important question is if *T. solium* eggs are abundant in the environment at the household levels in rural communities. Surprisingly little attention has been paid on the role of soil in transmission of *T. solium* cysticercosis. To date, research on soil contamination by soil-transmitted helminths has mainly focused on different nematodes of medical interest [14]. Although *T. solium* in soil has been screened for in the Iringa district of Tanzania, this study was based on standard microscopical examination [15]. Various staining techniques have been used to detect and differentiate between *Taenia* spp. eggs, but these suffer from limitations [16]. It was not until recently, a droplet digital Polymerase Chain Reaction (ddPCR) technique was validated for the detection of parasite egg DNA from soil [17]. However, application of this technique when performed in soils collected from the field environment has not been done.

In a previous study, we found a 17% seroprevalence rate of cysticercosis in pigs in rural villages of Kongwa district in Tanzania [5]. To improve the understanding of human and porcine cysticercosis infection-risk in more detail, it was decided to investigate the level of soil contamination by *T. solium* eggs at the village and household levels. In this study, we aimed to assess the rate of contamination by *T. solium* eggs in soils using the ddPCR approach to establish the risk of environmental *T. solium* transmission.

## Materials and methods

### Study area

**Figure 1.**
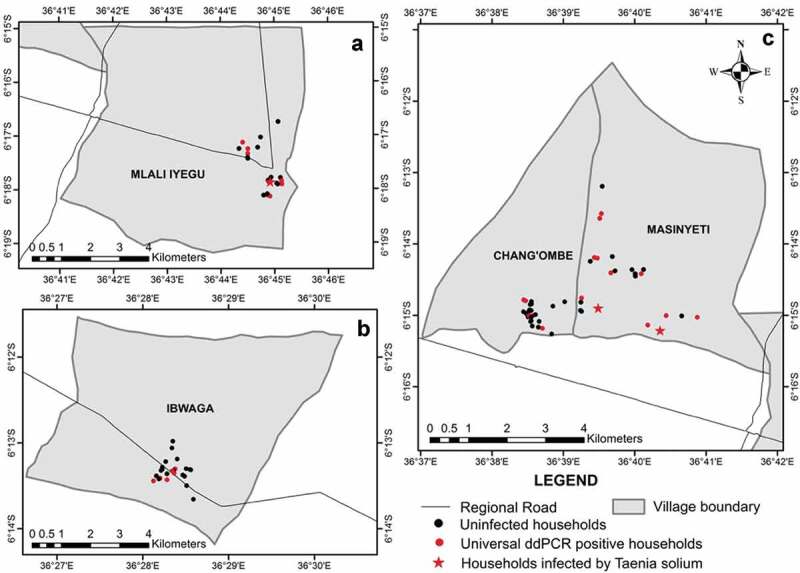
Villages in Kongwa district showing surveyed households. For the locations of the villages in Kongwa see 5. Black circles, red circles and red stars show soils from households identified as negative, universal ddPCR positive or positive for *T. solium*, respectively.

This study was conducted in four villages (Chang’ombe, Ibwaga, Masinyeti, and Mlali Iyegu) in the Kongwa district (5°30ʹ & 6°0’S and 36°15′ & 36°0ʹE) of the Dodoma region in eastern-central Tanzania (Figure 1). The population of Kongwa in 2012 was 309,973 people with an average of 4.9 persons per household with a projection to reach 343,975 people in 2017 (NBS, 2014; 18). The population of Chang’ombe, Ibwaga, Masinyeti and Mlali Iyegu villages were estimated to reach 4,846, 2,435, 3,430 and 12,613 people in 2017, respectively [NBS, 2014; 19]. The mean annual temperature ranges between 18°C to 34°C, while the annual mean rainfall is approximately 500 mm [20]. The villages were surveyed over two seasons: July – August 2017 (dry season) and March – April 2018 (rainy season). The household economy in Kongwa villages depends on the crop (92%) and livestock (38%) production [21]. Kongwa in comparison to other districts in the region, has the largest number of pigs (48%) with an average of four pigs per household [21], reared under the free and semi-intensive system. Dogs and cats are also common in many households. Like other areas in the southern and northern highlands of Tanzania, the main form of pig production in rural villages of Kongwa is under free-range conditions, though primitive housing is sometimes provided. In addition, most households in rural villages of Kongwa posses unimproved pit latrines [5].

### Soil sample collection

A total of 24 pig-keeping households were surveyed in each of the four villages. In both rain and dry seasons, soil samples weighing about 200 g each were collected approximately at the same locations from the surface to a maximum depth of 3 cm depending on the nature of the surface, using plastic spoons, from five sampling points in each household. The soil samples were collected in polythene bags from; i) areas around the toilet, ii) the backyard, iii) inside the house, iv) in front of the house and v) around the waste disposal area. All investigated households had un-cemented floor and therefore only the top soil was swept and collected from inside the houses. Samples were stored in a cooler box during field sample collection. Before samples were transported to the Swedish University of Agricultural Sciences in Sweden for DNA extraction and analysis, they were stored at 4°C in the Zoology Laboratory of the Sokoine University of Agriculture in Tanzania.

### Screening of soil

In the laboratory, approximately 100 g of each soil sample from all five sampling points per household was pooled and thoroughly mixed and filtered through 2 mm mesh metal sieve (W.S. Tyler, Ohio, USA). Then, about 10 g of the filtered soil from each household was transferred into a 50 ml Falcon tube for *T. solium* egg recovery using 15 ml of saturated zinc chloride solution (density 1.45 g/ml, Acros Organics, Geel, Belgium), basically in accordance with a protocol originally developed for detection of the related cestode *Echinococcus multilocularis* eggs in fox faeces [22,23], with minor modifications as described in 17.

DNA was extracted from sample pellets recovered from household soil using the QIAamp DNA Mini Kit (Qiagen, Hilden, Germany) according to the following procedure: First, the volume of the suspension was adjusted to 200 µl using distilled water. Second, alkaline lysis and neutralization procedures were performed on the sample pellets [22,23]. This was achieved by adding 25 µl 1 M potassium hydroxide (KOH, Fluka Chemie AG, Buchs, Switzerland) and 7 µl 1 M Dithiothreitol (DTT, National Diagnostics, Atlanta, USA) to the soil suspensions, which were then vortexed and incubated at 65°C for 15 min. After incubation, the samples (200 µl each) were centrifuged at 200xg for 10 sec, followed by the addition of 60 µl 2 M Tris-HCl (pH 8.4) (Sigma Aldrich, Stockholm, Sweden) and 2 µl concentrated HCl (12.4 N/≥ 37%) (Merck Schuchardt, Hohenbrunn, Germany) and mixed by vortexing. Third, centrifugation at 200xg for 10 sec preceded the addition of 200 µl buffer AL from the QIAamp DNA Mini Kit, 20 µl Proteinase K, mixing by vortexing and then incubation of the samples at 56°C for 10 min on a thermoshaker (Grant Bio, UK). After spinning down the samples, 50 µl chelex (50%) (Sigma Aldrich, Stockholm, Sweden) was added, mixed thoroughly and incubated a second time at 25°C for 3 h on the thermoshaker. After centrifugation at 6000xg for 1 min, the supernatant was transferred to a clean microcentrifuge tube. Finally, the DNA elution process proceeded as described in the QIAamp DNA Mini Kit [DNA purification from tissues) user manual. The extracted DNA was stored at −20°C in labelled microcentrifuge tubes before DNA analysis.

### DNA examination

Initially, all 192 samples were screened using universal primers suggested by 3, which amplify the mitochondrial cytochrome c oxidase subunit I (*cox1*] gene from a range of cestodes and trematodes, in ddPCR EvaGreen Supermix (Bio-Rad Laboratories) [universal ddPCR assay) as described in 17. The presence of *T. solium* DNA was then confirmed in all universal ddPCR assay positive reactions using a specific primers probe set in ddPCR Supermix for Probes with no dUTP (Bio-Rad Laboratories] (specific ddPCR probe assay) targeting the mitochondrial *cox1* gene of *T. solium*. Then, each of the five soil samples from each household (for which the pooled soil sample tested positive for *T. solium*) was analysed separately using the specific ddPCR probe assay to identify the location contaminated by the parasite egg DNA. The sequences for the universal primers (JB3 and JB4.5), *T. solium* specific primers (Tsol_F and Tsol_R) and probe (Tsol_probe) are shown in Table 1. Specific primers and probe were designed using Primer3Plus [24].

**Table 1.** Primer and probe names and sequences used in ddPCR assay.

Name	Description	Sequence (5ʹ to 3ʹ)	Reference
JB3	Forward	TTTTTTGGGCATCCTGAGGTTTAT	[25]
JB4.5	Reverse	TAAAGAAAGAACATAATGAAAATG	[25]
Tsol_F	Forward	ATTCTTCCGGGGTTTGGTAT	This study
Tsol_R	Reverse	CCTTAATCCCCGTAGGCACT	This study
Tsol_probe	FAM probe	56-FAM/TGTGTTCTGATGCTTTTGGC/31ABkFQ	This study

The optimal annealing temperature for the universal ddPCR assay was set to 54°C [17]. For the ddPCR probe assay, the annealing temperature was determined to be 56°C by a thermal gradient ranging from 50°C to 60°C with 2 µl of template *T. solium* cysticerci DNA extracted from pigs [5].

The ddPCR DNA template and reaction mixtures, 22 µl each, were dispensed into the wells of a 96-well plate (Sarstedt AG & Co. KG, Germany). The reaction mixture volume consisted of 11 μl 2x Supermix for Probes, 1 μL 20x of each primer at a concentration of 900 nM and 0.28 µl 20x probe (FAM) at a concentration of 250 nM, 6.72 μL molecular-grade H_2_O and 2 μl soil DNA template. In addition to the tested samples, we included a no template control (molecular-grade H_2_O) and *T. solium* DNA extracted from *T. solium* cysticerci from pigs as negative and positive controls, respectively. All samples were tested in technical duplicates. The QX200 Automated Droplet Generator (Bio-Rad Laboratories) was used to partition the samples into droplets, followed by heat- sealing of the plates containing the samples. Droplet generation was then followed by an amplification step in a MyCycler thermal cycler (Bio-Rad Laboratories) under the following thermal cycling conditions: Enzyme activation at 95°C for 5 min, followed by 40 cycles of denaturation at 94°C for 30 sec and annealing at 56°C for 1 min after which the enzyme was deactivated at 98°C for 10 min. Finally, results were read in a QX200 Droplet Reader (Bio-Rad Laboratories).

### Data analysis

QuantaSoft^TM^ version 1.7.4 software (Bio-Rad Laboratories) was used to analyse the number of positive and negative droplets generated in the Bio-Rad QX200 Droplet Reader. A threshold automatically set by the reader to the 1-D amplitude data distinguished positive from negative droplets. Manual thresholding was applied when droplet cluster adjustment was required. All reaction mixtures with or above 10,000 droplets were included in the analysis. Analysis of variance (ANOVA) was performed to compare *T. solium* mitochondrial *cox1* copies generated with the universal ddPCR assay and the *T. solium* specific probe ddPCR assay. When ANOVA results were statistically significant (*p* < 0.05), Bonferroni’s multiple comparisons *post hoc* test was used to compare the groups. Statistical data analysis employed Microsoft Excel 2016 for Windows (Microsoft Corporation, Redmond, Washington, USA) and GraphPad Prism version 8.3.0 for Windows (GraphPad Software, La Jolla California, USA).

## Results

### Soil screening results of ddPCR using universal ddPCR assay

A total of 192 pooled soil samples from 96 households in the four villages in Kongwa were screened for *T. solium* eggs contamination during the dry (n = 480) and rainy (n = 480) seasons. Using the universal ddPCR assay, 17.2% (n = 33/192) of the samples were deemed helminth DNA positive. The number of mitochondrial *cox1* gene copies detected ranged between 12 and 4,940 per well.

### Screening universal ddPCR positive reactions using specific ddPCR probe assay

DNA of *T. solium* was only confirmed in 9.1% (n = 3/33) of universal ddPCR assay positive reactions, which constituted 1.6% (n = 3/192) of the household soil analysed and 3.1% (n = 3/96) of the households surveyed. Thus, the overall prevalence of *T. solium* DNA contamination in household soils in the surveyed villages in Kongwa district during the two seasons was found to be low (1.6%). Contamination by *T. solium* (3.1%, n = 3/96) was only recorded in two villages during the dry season, whereas no contamination was recorded during the rainy season (n = 0/96). Contamination was detected in areas around the toilet (household MA3), waste disposal site (household MA6) and outside the house (household ML14) (Table 2).

**Table 2.** Statistical analysis of mean mitochondrial *cox1* gene copies per reaction mixture of *T. solium* detected both by universal (u) and specific (s) ddPCR assays in DNA samples extracted from household soil tested in duplicate.

Sample	uddPCR	sddPCR	MD	*p* value	uddPPR			sddPCR	
P25	16,946	19,940	2994	< 0.05	Sample pairs	MD	*p* valuea	MD	*p* valuea
MA3	41	29	12	> 0.05	MA3 vs MA6	94	> 0.05	107	> 0.05
MA6	135	136	1	> 0.05	MA3 vs ML14	22	> 0.05	24	> 0.05
ML14	63	53	10	> 0.05	MA6 vs ML14	72	> 0.05	83	> 0.05

a Bonferroni’s multiple comparisons test; MD = Mean Difference; MA3 & MA6 and ML14 = Households contaminated by *T. solium* DNA in Masinyeti and Mlali Iyegu villages, respectively; P25 = Positive control; uddPCR = universal ddPCR assay; sddPCR = specific ddPCR probe assay.

The number of mitochondrial *cox1* copies detected in all three *T. solium* positive reactions ranged between 29–136 and 41–135 per well for the specific and universal ddPCR assay, respectively.

### Comparison between the universal and specific ddPCR assays

On average, the universal ddPCR assay detected the highest total mean number (52%) of mitochondrial *cox1* gene copies per reaction mixture in the three *T. solium* positive households as compared to specific ddPCR probe assay (48%) (Table 2), although the difference was not significant (*F_1,2_* = 3.00, *p* > 0.05). Further, multiple comparisons of *T. solium* mean mitochondrial *cox1* gene copies per reaction mixture recovered from the three households using the specific ddPCR probe assay indicated no significant statistical difference (*F_2,6_ *= 4.29; *p* > 0.05).

## Discussion

This is the first-time soil contamination by *T. solium* eggs has been investigated in the field by the use of ddPCR. The study was conducted from the same households in four Tanzanian villages both during the rainy and dry seasons. A low level (3.1%) of soil contamination by *T. solium* egg DNA was detected using a specific ddPCR assay from the soil in the surveyed households. Parasite egg DNA was detected in areas around the toilet, waste disposal site and outside the house. Although research has been carried out on porcine and human cysticercosis in Tanzania [2,5,7], researches on soil contamination by *T. solium* eggs are scarce and none has been empirically examined with ddPCR.

To the best of our knowledge, this is the first study that has attempted to investigate *T. solium* eggs by detection of DNA isolated from household soils. Nevertheless, the findings of *T. solium* DNA in the soil in our study are lower than what has been reported for *E. multilocularis* in soil from northeast Poland [26], which utilized different methods for egg recovery and DNA detection. On the other hand, the results are consistent with those in a European study, that found both *Taenia* and *Echinococcus* eggs on vegetables and fruits [27], and differ from previous studies using microscopy, which were unable to detect *T. solium* eggs from soil in endemic areas [15,28,29]. The rate of soil DNA contamination by *T. solium* eggs in this study are also lower than those reported for other soil-transmitted helminths such as *Ascaris, Trichuris* and *Ancylostoma* [14]. A note of caution is due here since these results are based on various methods for recovery and detection of parasite eggs from various sources and in different environments.

Gravid proglottids of *T. solium* contain several thousands of eggs but they are non-motile, unlike those of *Taenia saginata* and *Taenia asiatica*, which account for an even more clumped distribution of eggs once in the environment [3]. Thus, the relatively low prevalence of *T. solium* DNA recorded from the soils in this study might be due to the heterogeneous distribution of the cestode eggs. That is assuming a skewed dispersion of the parasite eggs in the environmental soil, field sampling of the parasite eggs from soil become more probabilistic. The amount of soil analysed per household was relatively low and may have also contributed to the recorded low level of contamination. Although soils were collected from five sampling points per household, for logistic reasons these were pooled (5 x 100 g), from which a single sample of about 10 g was analysed in each season. This way, soils from only three households were confirmed to contain *T. solium* DNA. Thus, our sampling design might have underestimated the presence of *T. solium* eggs in the soil. The likelihood of detecting contamination of *T. solium* eggs in the household environment in endemic areas can probably be enhanced by sampling hotspots such as around remnants of deposited human faeces.

In a previous study, a high prevalence of taeniid antigens was recorded in pigs from the same villages in Kongwa [5], suggesting environmental contamination with *T. solium* eggs. This finding is not supported by the results presented herein. It seems like the high prevalence stems from the coprophagic nature of pigs, which at the same time might have contributed to a reduced rate of soil contamination by *T. solium* eggs. In summary, our observations suggest that pigs acquire cysticercosis mainly by the consumption of eggs present in human faeces rather than from contaminated soil. Previous studies from the same study area have shown that 98% of the households have pit latrines, many of which are unimproved [5]. In addition, open defecation by humans was reported, suggesting poor utilization of pit latrines, which give free-roaming pigs access to potentially infected human faeces and hence a risk of contracting cysticercosis. Education on the proper use of sanitary facilities is thus important to reduce the risk of infection for both pigs and humans.

In the present study, *T. solium* DNA in soil was exclusively detected in the dry season. Similarly, a lower rate of other soil-transmitted helminths’ eggs has been recorded in the rainy as compared to the dry season [30]. The absence of soil contamination by *T. solium* eggs in the rainy season might be because rainwater washes off the eggs into surface water, where they may contaminate rivers and streams [31]. In both seasons, the weight of each sample collected was equivalent (≈200 g), and thus the volume of soil collected during the rainy season was lower compared to the dry season. The difference in the volume of soil collected in both seasons might also explain the difference in the detection of *T. solium* DNA.

The ddPCR is obviously a promising technology for the assessment of *T. solium* eggs contamination in environmental samples due to its robustness against inhibitors and high sensitivity [32]. In addition, this technology generates results directly in the form of target gene copy numbers without a standard curve [33]. Our results indicate that the ddPCR assays used herein were effective (Table 2). However, it was also found that the direct application of ddPCR, without the need for confirmatory sequencing, requires the use of specific primers. A limitation with ddPCR for low-income countries is that the costs for equipment and consumables are high [33].

In conclusion, we report herein a low level of soil contamination by *T. solium* from the surveyed households in rural villages in Kongwa district, Tanzania. The recovery of *T. solium* DNA from soil indicates that ddPCR is a promising technology, which potentially can be useful for future environmental screening of *T. solium* eggs in endemic areas. Overall, our results suggest that the risk of acquiring a *T. solium* infection from the soil is low for both pigs and humans in the studied villages. There is still a need to investigate, in more detail, the most important focal points of soil contamination. It is also important to explore other environmental and ecological factors that pose risks to cysticercosis infection such as locally grown vegetables, pig feed or water sources. It would be also interesting to assess the effectiveness of ddPCR using other genetic markers such as repetitive elements in the ribosomal DNA gene (rDNA) array. Efforts to make the ddPCR technology affordable by low-income countries for future epidemiological studies are encouraged.
